# Is dietary or microhabitat specialization associated with environmental heterogeneity in horned lizards (*Phrynosoma*)?

**DOI:** 10.1002/ece3.5109

**Published:** 2019-03-28

**Authors:** Jeanine M. Refsnider, Sarah E. Carter, Gunnar R. Kramer, Adam D. Siefker, Henry M. Streby

**Affiliations:** ^1^ Department of Environmental Sciences University of Toledo Toledo Ohio

**Keywords:** Abajo Mountains, diet, ground cover, horned lizard, *Phrynosoma hernandesi*, shade cover, trophic level

## Abstract

Niche breadth is predicted to correlate with environmental heterogeneity, such that generalists will evolve in heterogeneous environments and specialists will evolve in environments that vary less over space and time. We tested the hypothesis that lizards in a heterogeneous environment were generalists compared to lizards in a homogeneous environment. We compared niche breadths of greater short‐horned lizards by quantifying resource selection in terms of two different niche axes, diet (prey items and trophic level), and microhabitat (ground cover and shade cover) between two populations occurring at different elevations. We assessed the heterogeneity of dietary and microhabitat resources within each population's environment by quantifying the availability of prey items, ground cover, and shade cover in each environment. Overall, our results demonstrate that despite differences in resource heterogeneity between elevations, resource selection did not consistently differ between populations. Moreover, environmental heterogeneity was not associated with generalization of resource use. The low‐elevation site had a broader range of available prey items, yet lizards at the high‐elevation site demonstrated more generalization in diet. In contrast, the high‐elevation site had a broader range of available microhabitats, but the lizard populations at both sites were similarly generalized for shade cover selection and were similarly specialized for ground cover selection. Our results demonstrate that environmental heterogeneity of a particular resource does not necessarily predict the degree to which organisms specialize on that resource.

## 1 INTRODUCTION

Niche breadth is hypothesized to correlate with environmental heterogeneity, such that generalists should be favored in heterogeneous environments, while specialists will have an advantage in more homogeneous environments that vary little over space and time (Kassen, 2002; Levins, 1968; Scheiner, 1993; Via & Lande, 1985). Resource specialists have behavioral, morphological, and/or physiological adaptations that allow them to efficiently exploit a particular resource, and which may indicate local adaptation to the resource in their particular environment (e.g., Taylor, Ternes, & Lattanzio, 2018). Resource generalists, in contrast, may have fewer adaptations specific to a local environment, and less stringent resource requirements than a specialist, allowing them to exploit a wider range of resources than a specialist. The ecological consequence of the specialist/generalist dichotomy is the “jack‐of‐all‐trades, master‐of‐none” tradeoff (Levins, 1968; MacArthur, 1972): under optimal and relatively constant conditions, specialists are expected to outcompete generalists because generalists must contend with the added costs of exploiting multiple resources and tolerating a wider range of environmental conditions (Richmond, Breitburg, & Rose, 2005). However, generalists may be better able to contend with environmental change than specialists (Clavel, Julliard, & Devictor, 2011; Rowe, Terry, & Rickart, 2011), and generalists may persist longer in sub‐optimal or degraded habitat (Richmond et al., 2005, but see Attum, Eason, Cobbs, & Baha El Din, 2006).

Where an organism falls along the specialist–generalist continuum varies among populations, and even among individuals within a population. For example, populations that have undergone ecological release, particularly as a result of colonizing new habitat, often demonstrate a wider niche breadth than their ancestral counterparts (Bolnick, Svänback, Araújo, & Persson, 2007; Des Roches, Brinkmeyer, Harmon, & Rosenblum, 2015; Refsnider, Des Roches, & Rosenblum, 2015). At a finer scale, individuals within a population may vary in their degree of specialization on a particular resource, such that different individuals function as generalists or specialists within the same environment (Bolnick et al., 2010, 2007; Kamath & Losos, 2016). Importantly, however, whether a population or individual is categorized as a “specialist” or a “generalist” may differ depending on the niche axis in question (e.g., Devictor et al., 2010; Futuyma & Moreno, 1988). For example, a butterfly species could be a specialist that feeds and oviposits on a particular plant species, yet simultaneously tolerates a wide range of temperatures. Such a species would be considered a specialist for one resource (i.e., host plant) but a generalist for another (i.e., thermal conditions; Dennis, Dapporto, Fattorini, & Cook, 2011).

Lizards have become important model organisms for studying niche breadth, and in particular, rapid evolution of niche breadth. Many lizards exhibit morphological differentiation related to prey type, such as head size and bite force, and in some cases feeding morphology can evolve relatively rapidly in lizards. For example, geckos on newly created islands increased their food‐niche breadth by adding large termites to their diet, and concomitantly evolved larger relative head sizes than their mainland counterparts (De Amorim et al., 2017). Similarly, in several lizard populations that experienced recent ecological release, diets of lizards in newly colonized habitat included harder prey items, and lizards had stronger bite forces and larger heads, than lizards in the source population (Des Roches et al., 2015). Habitat type and morphology are also tightly linked in lizards (e.g., Losos, Warheit, & Schoener, 1997; Mahler, Ingram, Revell, & Losos, 2013; Williams, 1983). For example, anoles living in urban environments used broader perches, had longer limbs, and more toe lamellae than conspecifics from more natural sites (Winchell, Reynolds, Prado‐Irwin, Puente‐Rolon, & Revell, 2016). If dietary and microhabitat niche breadths can diverge rapidly between source and founding populations, as these studies demonstrate, might niche breadth also differ between populations whose environments differ in resource heterogeneity?

We used greater short‐horned lizards (*Phrynosoma hernandesi*, formerly *Phrynosoma douglasii*) to test the hypothesis that a population in an environment with greater heterogeneity for a particular resource would exhibit a wider niche breadth (i.e., generalization) for that resource, compared to a population in an environment more homogeneous for the same resource, which should exhibit a narrow niche breadth (i.e., specialization) for that resource. *Phrynosoma hernandesi* is a high‐elevation species occurring in montane communities of the U.S. Great Basin, Colorado Plateau, and Mexican Highlands. Throughout its range, it is a common inhabitant of sagebrush scrublands and piñon‐juniper forest (Hodges, 2009). Horned lizards have evolved a range of behavioral and morphological adaptations for ant‐eating, or myrmecophagy, although the short‐horned lizard clade, in which *P. hernandesi* falls, is thought to specialize less on ants and instead consume more hard‐bodied prey items than other horned lizards (Meyers, Herrel, & Nishikawa, 2006).

We measured *P. hernandesi* dietary and microhabitat niche breadth by quantifying trophic level and resource selection for prey items, ground cover, and shade cover in two populations occurring at different elevations. We assessed the heterogeneity of dietary and microhabitat resources within each population's environment by quantifying the availability of prey items, ground cover, and shade cover in each environment. We predicted that lizards from the site with a wider range of available prey items (i.e., greater environmental heterogeneity) would exhibit a broader dietary niche breadth (i.e., more generalized diet) than lizards from the site with a narrower range of available prey items. Similarly, we predicted that lizards from the site with a wider range of microhabitats (i.e., greater environmental heterogeneity) would exhibit broader selection of microhabitat types (i.e., more generalized microhabitat use) than lizards from the site with a narrower range of available microhabitats.

## 2 MATERIALS AND METHODS

### 2.1 Study sites

We studied *P. hernandesi* at two sites on North Peak in the Abajo Mountains of southeastern Utah. The low‐elevation site was 2,080 m in elevation and was primarily a sagebrush scrubland surrounded by piñon‐juniper forest, while the high‐elevation site was 2,550 m and was primarily piñon‐juniper shrubland interspersed with sagebrush patches. Both sites were on east‐ or northeast‐facing slopes. Individual home range sizes average 1,218 m^2^ at the low‐elevation site and 643 m^2^ at the high‐elevation site, and individuals do not travel between sites even when reciprocally transplanted (Refsnider et al., 2018). We captured lizards by hand and housed them individually in plastic terraria (Kritter Keepers; LLL Reptile and Supply Company, Inc., Oceanside, CA, USA) with sand substrate and a handful of local vegetation for shelter. All lizards were transported to our field lab at Canyonlands Research Center, where they were housed overnight at 24–28°C under ambient lighting. We determined sex based on the presence of enlarged postanal scales and orange femoral pores in adult males, and we palpated all females to ascertain gravidity.

### 2.2 Quantification of diet

We compared dietary niche breadth between low‐ and high‐elevation lizards in two ways. First, we compared prey items eaten by lizards to prey items available in the landscape to determine which prey items lizards selected or avoided at each site during the sampling period, which allowed us to estimate the breadth of prey items lizards consumed at each site. To quantify prey use, upon initial capture, we flushed the stomach of each lizard and collected the contents following the methods of Des Roches et al. (2015). Briefly, we induced the lizard to bite a plastic ring, which served to hold its jaws open while we gently inserted a dosing cannulus down its throat. Once the cannulus reached the stomach, we gently pumped 5 ml of lukewarm water into the cannulus using a syringe. This induced the lizard to regurgitate its food bolus, which was collected in a petri dish and subsequently stored in 70% EtOH. To assess the environmental heterogeneity at each study site for prey resources, we quantified the prey items available in each population's environment by sampling invertebrates using pitfall traps. At each study site, we used six, 50‐ml Falcon tubes as pitfall traps set every 10 m along a 50‐m transect. Each transect started within a piñon‐juniper patch and ended in an open sagebrush meadow, with the center of the transect located at the ecotone between forest and sagebrush habitat. Pitfall traps contained 10 ml of 70% EtOH to preserve captured invertebrates and were set between 8 and 11 August 2015; following retrieval, we stored the contents of each pitfall trap in fresh 70% EtOH. We subsequently sorted stomach (i.e., “used” prey items) and pitfall trap (i.e., “available” prey items) contents by taxonomic order, and counted the number of prey items in each order. We counted only intact or partially digested prey items in the stomach samples, and did not count individual body parts such as detached legs that could not readily be assigned to taxonomic order; these generally made up a very small proportion of the stomach contents.

As our second method of assessing dietary niche breadth, we compared the trophic level at which lizards from low‐ and high elevations were feeding using carbon (δ^13^C) and nitrogen (δ^15^N) stable isotope analysis. Stable isotopes provide a longer‐term assessment of diet, whereas stomach samples are a snapshot assessment of diet over the previous few days (Araújo, Bolnick, Machado, Giaretta, & Reis, 2007). To determine trophic levels of low‐ and high‐elevation lizards, we collected fecal pellets from each lizard's terrarium after lizards had been housed overnight. Fecal samples have previously been used to infer temporal changes in the diets of songbirds (Podlesak, McWilliams, & Hatch, 2005), and the large size of *Phrynosoma* fecal pellets relative to body size, combined with prey items containing substantial undigestable material, makes fecal samples ideal for estimating trophic position in this taxon. We also collected samples of eight common plant species at each site by clipping off new shoot growth. Fecal and plant samples were dried in a drying oven at 37°C for 48 hr, and were then ground to a fine powder. All samples were analyzed at the Colorado Plateau Stable Isotope Laboratory at Northern Arizona University using an isotope ratio mass spectrometer.

### 2.3 Microhabitat use

We compared microhabitat niche breadth between low‐ and high‐elevation lizards by comparing the ground cover types and amount of shade cover at sites used by lizards to ground cover and shade cover available in the landscape to determine which microhabitats lizards selected or avoided at each site during the sampling period. Ground cover was used here as an analog to “perch type,” which is commonly used to characterize microhabitat use in more arboreal lizards (e.g., Des Roches, Robertson, Harmon, & Rosenblum, 2011; Losos & DeQueiroz, 1997; Refsnider et al., 2015). Upon initial capture, we attached a 0.35‐g radio‐transmitter (Blackburn Transmitters, Inc., Nagadoches, TX, USA) to each lizard by gluing it directly to the dorsal scales using fast‐drying superglue (LocTite Super Glue Gel Control; described in detail in Refsnider et al., 2018). Following their release at the site of capture, all lizards were radio‐tracked daily during 4–13 August 2015. Each time we located a lizard via telemetry, we first recorded its location using a handheld GPS unit. We then set a camera fitted with a 180^o^‐fisheye lens directly on the ground, pointing upward, at the location where the lizard was first observed and took a hemispherical photograph to record the amount of shade cover over the site used by the lizard. We took a second photograph at the same location (without the fisheye lens), this time from a height of 1.5 m above the ground and pointing downward, to record ground cover at the site used by the lizard. To assess the environmental heterogeneity in microhabitat at each study site, we quantified the ground cover types and amount of shade cover available in each population's environment. Following each radio‐location of a lizard, we walked 10 m in a random direction from the lizard's location and took a second set of photographs as described above to record ground cover and shade cover at an unused but available location in the environment. Throughout this study, we followed ASIH guidelines for use of live reptiles in Field Research (American Society of Ichthyologists and Herpetologists (ASIH) 2004).

### 2.4 Statistical analyses

To compare dietary niche breadth between lizards at the two study sites, we first calculated selection indices for each category of prey item at each study site (Manly, McDonald, & Thomas, 1993). For each category of a resource, such as prey item taxonomic order, the selection index is a ratio of that category's use in proportion to its overall availability, such that a resource type used more than would be expected based on its availability is considered to be selected for, and a resource type used less than expected based on availability is selected against. We then used separate log‐linear models, with a Bonferroni adjustment for multiple comparisons (*m* = 8), to determine whether lizards overall were using prey taxa randomly (i.e., in accordance with their availability), or selecting them nonrandomly at each study site. We also used a log‐linear model to determine whether lizards selected prey taxa differently at the low‐ versus high‐elevation site. Finally, we used the invertebrates collected in pitfall traps to calculate the Shannon diversity index (Shannon, 1948) for available prey taxa at each study site as an indicator of relative environmental heterogeneity of prey resources between the two sites.

We tested for population differences in mean δ^13^C and mean δ^15^N between low‐ and high‐elevation lizards using *t* tests, and we tested for population differences in variances in δ^13^C and δ^15^N between low‐ and high‐elevation lizards using Levene's tests. We calculated trophic position of each low‐ and high‐elevation lizard as described in Des Roches, Harmon, and Rosenblum (2016). Trophic position was calculated using the following formula (from Post, 2002):

$$\text{Trophic position}=\lambda +\left(\delta^{15}N_{\mathrm{lizard}}-\delta^{15}N_{\mathrm{base}}\right)/\Delta_{n}$$

where *λ* = 1, the trophic position of the primary producers used to estimate δ^15^N_base_; δ^15^N_base_ was measured directly for eight plant species and pooled at each site (pooled mean δ^15^N_base_ for the low‐elevation site = 0.12; high‐elevation site = −0.51); δ^15^N_lizard_ was measured directly for each individual lizard; and Δ_n_ = 3.4‰ following averages determined from food chains in comparable lizard studies (e.g., Des Roches et al., 2016).

We quantified the percent ground cover at each used and available location from the downward‐facing photographs by visually estimating, to the nearest 10%, the percent of each photo made up by bare ground, ant mounds, woody debris, rocks, grass or forbs, shrubs or trees, and sagebrush. Because ground covered by trees or shrubs and sagebrush could overlap with the other ground cover categories, the total percentage of ground coverage was sometimes >100%. We then calculated selection indices for each ground cover category at each study site as described above for prey items. We used separate log‐linear models, with a Bonferroni adjustment for multiple comparisons (*m* = 6), to determine whether lizards used ground cover types randomly (i.e., in accordance with availability) or selected among ground cover types nonrandomly at each study site. We also used a log‐linear model to determine whether lizards selected ground cover types differently at the low‐ versus high‐elevation site. Finally, we used availability of the different ground cover types at unused locations to calculate the Shannon diversity index (Shannon, 1948) for ground cover types at each site as an indicator of relative environmental heterogeneity of ground cover microhabitat between the two sites.

We quantified the percent shade cover over each used and available location from the hemispherical photographs using Gap Light Analyzer (Frazer, Canham, & Lertzman, 1999). Locations were then classified into one of five bins based on percent shade cover (0%–20%, 21%–40%, 41%–60%, 61%–80%, or 81%–100% shade cover). We calculated selection indices for each shade cover bin at each study site, and used separate log‐linear models, with a Bonferroni adjustment for multiple comparisons (*m* = 5), to determine whether lizards used shade cover randomly or selected it nonrandomly at each site, as described above. We used a log‐linear model to determine whether lizards at each site selected shade cover differently, and we calculated Shannon diversity indices for available shade cover at each site, as described above.

## 3 RESULTS

### 3.1 Dietary breadth: Prey item selection

**Figure 1:**
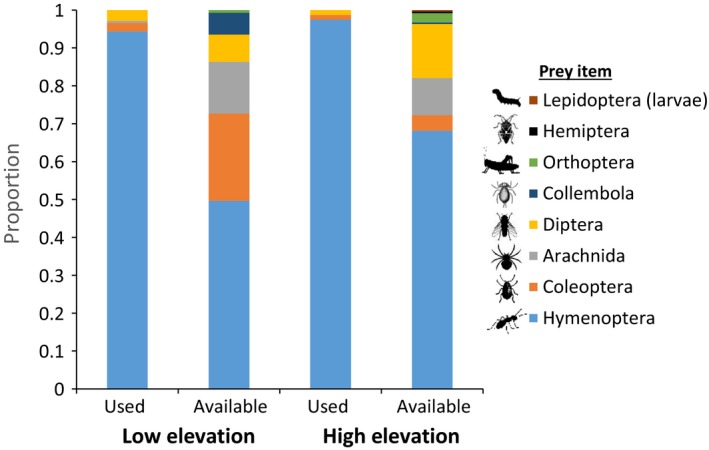
Proportion of invertebrate orders in greater short‐horned lizard (*Phrynosoma hernandesi*) stomach samples (i.e., used prey items) and pitfall traps (i.e., available prey items) at low‐ and high‐elevation sites on North Peak, Abajo Range, San Juan County, Utah in August 2015

We collected stomach contents from 17 low‐ and 11 high‐elevation lizards, and we sampled prey item availability for 18 trap‐days at each site. At both sites, ants (Hymenoptera) made up the highest percentage of items found in stomach contents (low site = 94%; high site = 97%), with a small proportion of stomach contents at both sites also including beetles (Coleoptera) and flies (Diptera; Figure 1). Ants were also the highest percentage of available prey items sampled in the pitfall traps at both sites (low site = 50%; high site = 68%). At the low‐elevation site, beetles (23%) and spiders (Arachnida; 13%) were the next‐most commonly sampled available prey items, whereas at the high‐elevation site flies (14%) and spiders (10%) were the most commonly sampled available prey items following ants. Overall, the low‐elevation site had a slightly higher diversity of available prey items than the high‐elevation site (Shannon diversity index, low site = 1.35; high site = 1.06).

Lizards selected prey items nonrandomly at both the low‐ (*df* = 5, *χ* ^2^ = 237, *p* < 0.001) and high‐elevation (*df* = 7, *χ* ^2^ = 240, *p* < 0.001) sites. Prey selection also differed between the two sites when controlling for differences in availability of prey items between the sites (*df* = 1, *χ* ^2^ = 4.67, *p* = 0.031). At both sites, lizards selected for ants; however, at the high‐elevation site, lizards also selected for beetles, whereas at the low‐elevation site they selected against beetles when accounting for beetles’ availability in the environment.

### 3.2 Dietary breadth: Trophic position

**Figure 2:**
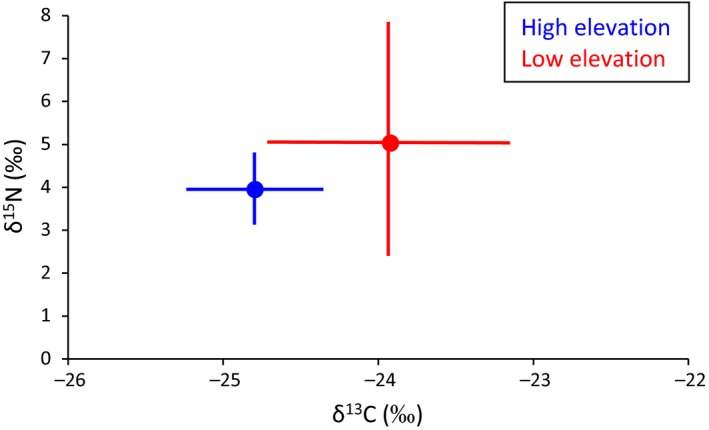
Pooled mean and standard deviation of δ^13^C and δ^15^N for greater short‐horned lizards (*Phrynosoma hernandesi*) at low‐ (red) and high‐elevation (blue) sites on North Peak, Abajo Range, San Juan County, Utah in August 2015

We measured δ^13^C and δ^15^N content in fecal pellets from 11 low‐ and six high‐elevation lizards. Mean δ^15^N did not differ between lizards from the two sites (*t* = −0.93, *p* = 0.36), but δ^13^C was higher for lizards from the low‐elevation site compared to the high‐elevation site (*t* = −2.52, *p* = 0.02), indicating differences in the composition of the primary producers between the two sites. Variances in δ^15^N and δ^13^C did not differ between sites (*F*_1,15_ = 0.77, *p* = 0.40; *F*_1,15_ = 1.04, *p* = 0.32, respectively). Lizards at both sites fed at a similar trophic level (mean, low site = 2.4 ± 0.8; mean, high site = 2.3 ± 0.24; *t* = −0.38, *p* = 0.71; Figure 2).

### 3.3 Microhabitat breadth: Ground cover type

**Figure 3:**
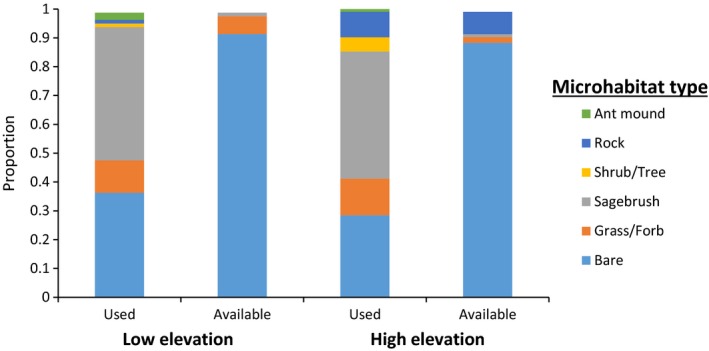
Proportion of ground cover types at points occupied by greater short‐horned lizards (*Phrynosoma hernandesi*; i.e., used ground cover types) and at available but unused points (i.e., available ground cover types) at low‐ and high‐elevation sites on North Peak, Abajo Range, San Juan County, Utah in August 2015

We collected data on ground cover type and amount of shade cover at 80 pairs of lizard (used) and random (available) locations at the low‐elevation site and 100 pairs of locations at the high‐elevation site. At both sites, bare ground made up the highest percentage of available ground cover types (91% and 88% of sampled available locations at the low‐ and high‐elevation sites, respectively), followed by grass and forbs at the low‐elevation site (6%) and rocks at the high‐elevation site (8%). The percentage of sampled available locations at each site that was sagebrush was only 10%. However, sagebrush constituted 46% and 44% of ground cover types used by lizards at the low‐ and high‐elevation sites, respectively (Figure 3). Overall, the high‐elevation site had a higher diversity of available ground cover types than the low‐elevation site (Shannon diversity index, low site = 0.38; high site = 0.49).

Lizards selected ground cover types non‐randomly at both the low‐ (*df* = 6, *χ* ^2^ = 28.43, *p* < 0.001) and high‐elevation (*df* = 6, *χ* ^2^ = 34.81, *p* < 0.001) sites. However, ground cover selection did not differ between the two sites after controlling for differences in availability of ground cover types between the sites (*df* = 1, *χ* ^2^ = 0.23, *p* = 0.48). At both sites, lizards showed selection for sagebrush and against all other ground cover types.

### 3.4 Microhabitat breadth: Amount of shade cover

**Figure 4:**
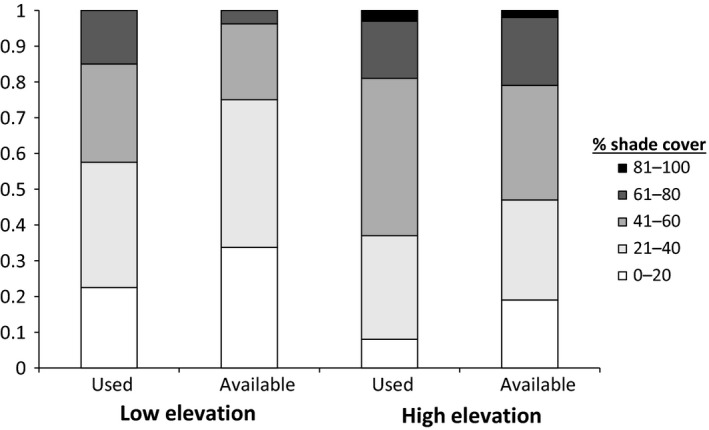
Proportion of points in ten different categories of shade cover occupied by greater short‐horned lizards (*Phrynosoma hernandesi*, i.e., used shade cover) and at available but unused points (i.e., available ground cover types) at low‐ and high‐elevation sites on North Peak, Abajo Range, San Juan County, Utah in August 2015

There was a wider range of shade cover available at the high‐elevation site than at the low‐elevation site. Locations with 21%–40% shade cover made up the highest proportion of sampled available locations at the low‐elevation site (41%), followed by locations with 0%–20% shade cover (34%). No locations at the low‐elevation site had 81%–100% shade cover, and locations with 61%–80% shade cover made up only 4% of sampled available points at the low‐elevation site. In contrast, at the high‐elevation site, the highest proportion of sampled available points had 41%–60% shade cover (32%), followed by 21%–40% shade cover (28%). Higher amounts of shade cover were more commonly available at the high‐elevation site, with 21% of sampled available points including >60% shade cover, including 2% of locations with 81%–100% shade cover (Figure 4). Overall, the high‐elevation site had a higher diversity of available shade cover than the low‐elevation site (Shannon diversity index, low site = 1.18; high site = 1.43).

Use of shade cover by lizards followed availability in the environment at both sites (*p*‐values >0.05). Lizards at both sites tended to select for greater amounts of shade cover, with lizards at the low‐elevation site selecting for 61%–80% shade cover and against other amounts; high‐elevation lizards selected for 41%–60% and 81%–100% shade cover, were neutral with respect to 21%–40% shade, and selected against 0%–20% and 61%–80% shade. Overall, lizard selection of shade cover did not differ between the low‐ and high‐elevation sites (*df* = 1, *χ* ^2^ = 0.07, *p* = 0.47; Figure 4) when controlling for differences in shade cover availability between sites.

## 4 DISCUSSION

Heterogeneous environments are predicted to support organisms that exhibit a more generalized strategy of resource use than are more homogeneous environments (Futuyma & Moreno, 1988; Kassen, 2002). This prediction arises because environments that vary, sometimes unpredictably, over space and time likely favor individuals with a wider niche breadth that can exploit a wider variety of resources, as compared to specialists constrained to a narrower set of resources which may not always be available in a variable environment (Gilchrist, 1995; Levins, 1968). In experimental evolution studies, selection acting in variable environments results in the evolution of generalists, while selection acting in constant environments results in the evolution of specialists in a variety of taxa, including viruses (Weaver, Brault, Kang, & Holland, 1999), bacteria (Bennett, Lenski, & Mittler, 1992), algae (Reboud & Bell, 1997), and insects (Janzen, 1973). Importantly, generalist and specialist strategies each entail costs which may constrain evolution of niche breadths. On one hand, specialists incur costs of adaptation, particularly antagonistic pleiotropy and accumulation of mutations that are deleterious in all but the specialist's home environment (Kawecki, Barton, & Fry, 1997; Travisano & Lenski, 1996). On the other hand, the broad environmental tolerance of generalists may result in negative fitness correlations across environments, such that generalists become jack‐of‐all‐trades but are masters of none (Futuyma & Moreno, 1988; MacArthur, 1972). The potential costs of specialization and generalization have important conservation implications because specialized populations are predicted to be more vulnerable than generalists to habitat fragmentation and degradation (Clavel et al., 2011; but see Jacob et al., 2018). Nevertheless, if niche breadth evolves to match the magnitude of environmental variation, then environmental heterogeneity may be critical in maintaining diversity at both the genetic and species levels (Kassen, 2002).

We tested the hypothesis that niche breadth would be wider, indicating greater generalization in resource use, in a more heterogeneous environment, while a more homogeneous environment would support more specialization in resource use, as demonstrated by a narrower niche breadth. Although resource heterogeneity and niche breadth differed between our study sites, our results provided no support for the prediction that the population in the heterogeneous environment would demonstrate more generalized resource use, as measured by wider niche breadth, than the population in the homogeneous environment. We found that, despite the low‐elevation site having a modestly broader range of available prey items and therefore representing a more heterogeneous environment for prey resources than the high‐elevation site, lizards at the high‐elevation site had a more generalized diet as demonstrated by a modestly wider niche breadth for prey items (although lizards at both sites fed at similar trophic levels). In contrast, the high‐elevation site had a broader range of available microhabitats, in terms of both ground cover types and range of shade cover, and therefore was a more heterogeneous environment for microhabitat, than the low‐elevation site. However, the two lizard populations demonstrated a similar degree of generalization for amount of shade cover and a similar degree of specialization for ground cover type (i.e., sagebrush), as indicated by the populations’ similar niche breadths for both axes of microhabitat.

While we found greater heterogeneity in ground cover types at the high‐elevation site, lizards in both populations showed strong selection for sagebrush, demonstrating specialization for this particular type of ground cover. At a very fine spatial scale, sagebrush provides a variety of microhabitats: the stiff and dense branches provide cover and protection against predators, while small open patches between branches allow lizards to bask in full sun while still sheltering within the bush itself. We have occasionally observed *P. hernandesi* climbing up into low branches of sagebrush plants to bask (Refsnider et al., 2018), but we have not observed this behavior by *P. hernandesi* in other shrub or bush species, likely because their lowest branches are too high off the ground for horned lizards, a primarily terrestrial clade, to access. In contrast to lizards’ strong selection for a particular ground cover type, we found that lizards at both the low‐ and high‐elevation sites used shade cover in accordance with its availability. Thus, lizards did not exhibit specialization for a particular range of shade cover at either study site. Previous research has demonstrated that lizards thermoregulate more effectively when their preferred microhabitats are dispersed rather than clumped in space (Sears et al., 2016). We did not assess spatial dispersion of shade cover in our study, but it is possible that lizards’ use of shade cover in accordance with its availability at both study sites reflects thermoregulatory behavior across a landscape in which shade cover is relatively evenly dispersed. Importantly, amount of shade cover is known to be actively selected in other reptiles (Refsnider & Janzen, 2012), so the generalized use of shade cover we observed here likely reflects actual “choices” made by lizards and is not simply an artifact of random walks through available microhabitat. Our study did not include replicate sites at each elevation, which would be necessary to test the hypothesis that spatial dispersion of resources, rather than overall resource availability, could affect resource use and thereby degree of resource generalization. Additionally, we cannot draw conclusions regarding the relationship between elevation and resource use, or between resource availability and resource use more generally, without replicate study sites.

It is not uncommon for populations that have undergone ecological release to evolve greater generalization in resource use, often resulting from relaxation of competition and/or predation pressure (e.g., Bolnick et al., 2007; Des Roches et al., 2015; Refsnider et al., 2015). Similarly, populations raised in environments that fluctuate in conditions such as temperature or pH also tend to evolve greater generalization in tolerance to those conditions (e.g., Reboud & Bell, 1997, Hughes, Callum, & Bennett, 2007, Ketola et al., 2013, but see Condon, Cooper, Yeaman, & Angilletta, 2013). However, we did not find evidence in our system that heterogeneity in availability of diet and/or microhabitat resources per se was associated with degree of generalization, as measured by resource selection. Instead, between‐population differences in selection of prey items may be driven by niche partitioning to avoid interspecific competition, while selection of microhabitat did not differ between populations despite differences in availability of microhabitat. It is also possible that resource use by lizards, or resource availability in the environment, vary seasonally, such that environmental heterogeneity or organisms’ degree of generalization change over time. As we only measured resource use and availability during a limited timeframe, we are unable to assess temporal variation in resource heterogeneity or degree of generalization.

In support of the hypothesis that generalists are favored over specialists in spatially or temporally variable habitats, several studies have found that more generalist populations that evolved under fluctuating environmental conditions had reproductive success equal to or greater than specialists under a wide range of conditions (Condon et al., 2013; Weaver et al., 1999). For example, lineages of *Paramecium* raised under fluctuating temperature environments evolved into generalists, and produced more offspring, than the specialist lineages that evolved under constant temperature environments (Duncan, Fellous, Quillery, & Kaltz, 2011). We did not assess reproductive fitness in our study populations, but a logical next step in understanding local adaptation in these populations would be to conduct a reciprocal transplant experiment to compare resource use and subsequent fitness of lizards in a novel environment. It may be that phenotypic plasticity for resource use, rather than resource use itself, is under selection and lizards’ ability to alter resource use in response to novel conditions is the critical determinant of their survival and reproductive fitness (e.g., Jacob et al., 2018; Refsnider et al., 2018). If this were the case, then magnitude of plasticity in resource use as conditions vary over space and time may be a better descriptor of whether a population is generalized or specialized (e.g., Condon et al., 2013), and whether degree of generalization is correlated with spatial and temporal environmental heterogeneity, than a snapshot of resource use over a brief period during which resource availability is unlikely to vary.

Niche theory predicts that heterogeneous environments should favor generalists, while more homogeneous environments will favor specialists. We found that, despite differences in prey and microhabitat heterogeneity at two different sites, prey and microhabitat selection by horned lizards did not consistently differ between populations at those sites. Moreover, environmental heterogeneity was not associated with generalization of lizards’ resource use. Our results demonstrate that environmental heterogeneity of a particular resource does not necessarily predict the degree to which organisms specialize on that resource. Future research should consider whether magnitude of plasticity for resource use, rather than resource use itself, is correlated with environmental heterogeneity.

## CONFLICT OF INTEREST

None declared.

## AUTHOR CONTRIBUTIONS

JMR conceived the study. JMR, SEC, GRK, ADS, and HMS carried out field data collection. JMR, SEC, and ADS analyzed the data. JMR wrote the manuscript.

## Data Availability

Data are available from the Dryad Digital Repository (https://doi.org/10.5061/dryad.2kf78h8).
